# The accuracy of species-specific allometric equations for estimating aboveground biomass in tropical moist montane forests: case study of *Albizia grandibracteata* and *Trichilia dregeana*

**DOI:** 10.1186/s13021-019-0134-8

**Published:** 2019-12-19

**Authors:** Damena Edae Daba, Teshome Soromessa

**Affiliations:** Center for Environmental Science, College of Natural and Computational Sciences, Addis Ababa University, Addis Ababa, Ethiopia

**Keywords:** Afromontane rainforest, Model comparisons, Scatter plots, Semi-destructive, Species-specific

## Abstract

**Background:**

Application of allometric equations for quantifying forests aboveground biomass is a crucial step related to efforts of climate change mitigation. Generalized allometric equations have been applied for estimating biomass and carbon storage of forests. However, adopting a generalized allometric equation to estimate the biomass of different forests generates uncertainty due to environmental variation. Therefore, formulating species-specific allometric equations is important to accurately quantify the biomass. Montane moist forest ecosystem comprises high forest type which is mainly found in the southwestern part of Ethiopia. Yayu Coffee Forest Biosphere Reserve is categorized into Afromontane Rainforest vegetation types in this ecosystem. This study was aimed to formulate species-specific allometric equations for *Albizia grandibracteata* Tuab. and *Trichilia dregeana* Sond. using the semi-destructive method.

**Results:**

Allometric equations in form of power models were developed for each tree species by evaluating the statistical relationships of total aboveground biomass (TAGB) and dendrometric variables. TAGB was regressed against diameter at breast height (D), total height (H), and wood density (ρ) individually and in a combination. The allometric equations were selected based on model performance statistics. Equations with the higher coefficient of determination (adj.R^2^), lower residual standard error (RSE), and low Akaike information criterion (AIC) values were found best fitted. Relationships between TAGB and predictive variables were found statistically significant (p ≤ 0.001) for all selected equations. Higher bias was reported related to the application of pan-tropical or generalized allometric equations.

**Conclusions:**

Formulating species-specific allometric equations is found important for accurate tree biomass estimation and quantifying the carbon stock. The developed biomass regression models can be applied as a species-specific equation to the montane moist forest ecosystem of southwestern Ethiopia.

## Background

The tropical forest ecosystem has been playing a significant role in mitigating the atmospheric carbon dioxide concentration and associated climate change impacts. Particularly, this ecosystem is known for its highest carbon pool when compared to other biomes of the world [1–3]. It is the most productive ecosystem accounting for over 60% of global terrestrial photosynthesis and one-third of global primary productivity [4]. Generally, the accumulation of a great deal of carbon stock in the aboveground biomass of tropical forests was verified [5, 6]. However, the biomass information is uncertain for many tropical forests due to the paucity of site-specific allometric equations [5, 7]. Therefore, applying a robust method for carbon stock estimation is a crucial step for the successful implementation of climate change mitigation strategies like REDD+ [8].

Allometric equations are important for their application to local and national forest carbon assessments, as well as for global carbon balance assessments [9]. Primarily, the current issue of global carbon cycles is the prominent factor for the formulation of biomass regression models [7, 10, 11]. As a result, generalized pantropical allometric equations were developed by many researchers [5, 7, 12, 13]. The development of a generalized allometric equation was approached by measuring multiple tree species and it was intended to be applied to a broad range of tropical forests [12]. However, a great error is generated related to adopting generic pantropical allometric equations for many forests [14, 15]. Biomass error that can be generated at individual tree level is also regularly propagated bias at forest stand and country-level during the assessment of biomass and carbon stock change when the appropriate allometric equation is not used.

Environmental variations among different forests are the ultimate factors for the variation of their biomass. Climatic regimes are the prominent factors that affect the growth of woody plants and biomass accumulation of different forest stands [16, 17]. Also, environmental variability in the context of physiographic and edaphic conditions plays a significant role in the variation of species composition and biomass difference among different forest sites [18, 19]. Within-stand variation of biomass for different tree species is related to tree architecture, growth strategies and its dynamic interplay with the biophysical environments [20–22]. The difference of TAGB across a forest landscape is mostly related to the variation in slope, elevation, and aspect [18, 23]. Generally, tropical forests are known for their high diversity of woody plants. The application of multispecies pan-tropical equations to individual tree species generates uncertainty of TAGB [9, 24]. Therefore, formulating a species-and site-specific biomass regression model was found the best approach to accurately quantify biomass and carbon storage of forests [25–27].

Yayu Coffee-Forest Biosphere has comprised Afromontane Rainforest Vegetation types, which is home for the endemic *Coffea arabica* in Southwestern Ethiopia [28]. This forest is known for storing a good deal of carbon stock and high species diversity. According to [29], the forest is categorized into Eastern Afromontane Biodiversity Hotspots which has global significance. In Ethiopia, forestry and agricultural sectors are the major sources of carbon dioxide CO_2_ emission, contributing more than 85% of the country. The forestry sector alone contributes a total emission of about 37% CO_2_ emission in the country. The total contribution of other sectors like power, transport, industry, and buildings is less than 15% [30, 31].

To reduce the current rate of carbon dioxide emission, the country has devised forestry-based strategies like REDD+ in potential forest areas. However, the estimation of biomass and carbon stock change depends on generic pan-tropical allometric equations, which generate bias when applied to individual tree species [28]. Species-specific equations were not formulated for many tree species in Ethiopia. Therefore, this study is intended to formulate species-specific TAGB allometric equations considering selected tree species (*A. grandibracteata* and *T. dregeana).* The selected tree species have a wide range of ecological distribution across different parts of Africa [32]. *A. grandibracteata* species has multiple socio-economic benefits and ecological services. It is fast-growing species on forest soils with high moisture-holding capacity. *A. grandibracteata* is a medium-sized deciduous tree with a straight trunk to 20 m, and a flattened or layered crown [33]; it can grow up to 30 m [34]. *T. dregeana* is also a very large evergreen tree to 30 m, with a large straight trunk dividing into large branches and a rounded crown [32]. These attributes of the selected species are important for storing a high amount of biomass and carbon. The semi-destructive method of data collection was found environmentally sound approach in the situation of the biosphere reserve. The core and buffer zones of the Yayu coffee-forest biosphere reserve were established mainly for scientific research, biodiversity conservation, and for monitoring the ecological processes.

## Methods

### Site description

**Fig. 1:**
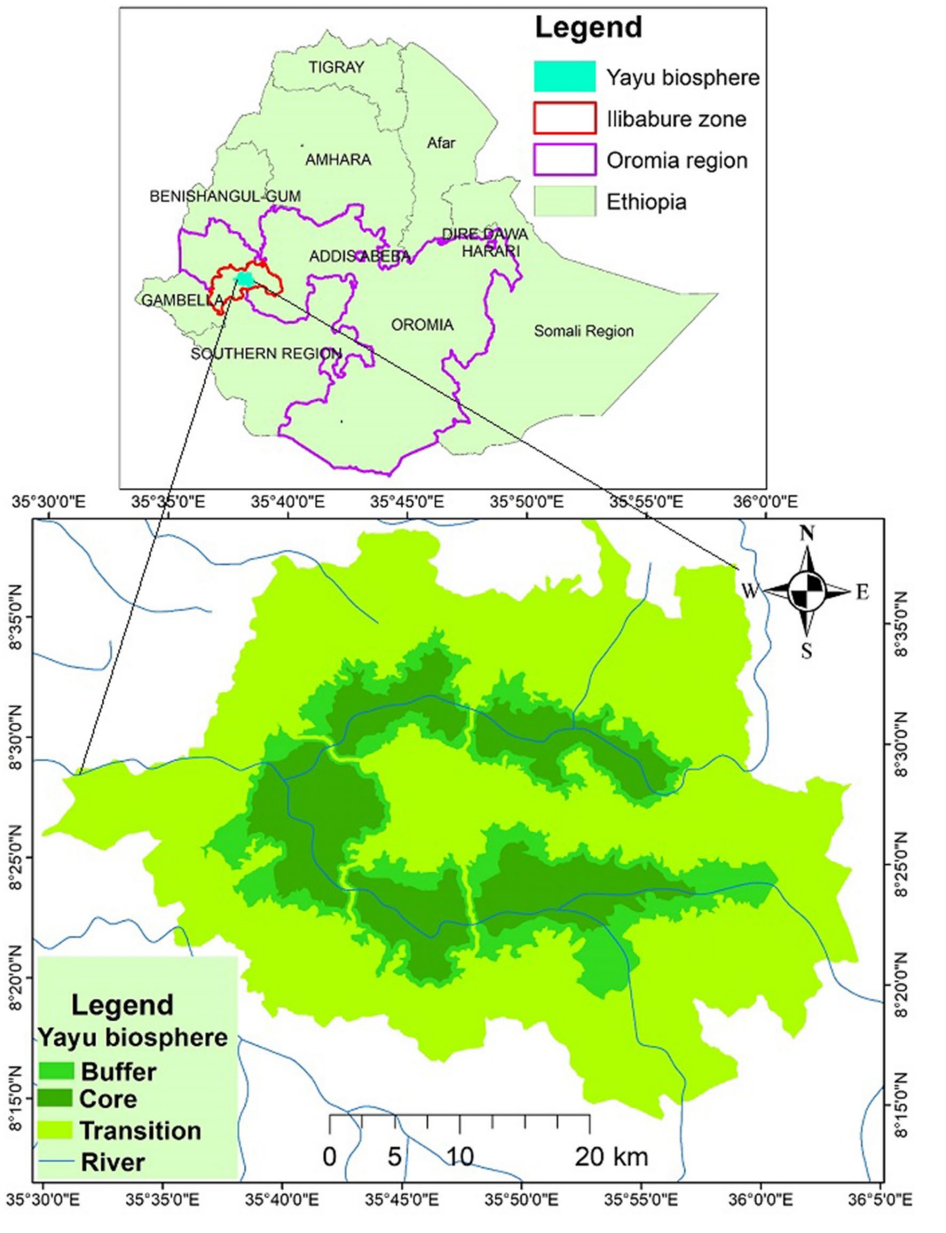
Map of Ethiopia with Oromia region, and the study area. The study area is marked green and the lines with blue color are rivers

The study was conducted in Yayu Coffee-Forest Biosphere Reserve which is located in Illubabor Zone, southwestern Ethiopia. The biosphere extends between latitude 8° 15′ 0′′–8° 35′ 0′′ N and longitude 35° 30′ 0′′–36° 0′ 0′′ E of zone 36 (Fig. 1). Detailed information regarding the study area extent, soil types, climatic condition, altitudinal range, and establishment of the biosphere reserve can be found in [28]. *Celtis africana*,* Diospyros abyssinica*,* Albizia grandibracteata*,* Ehretia cymosa*,* Trichilia dregeana*,* Vangueria apiculata. Argomuellera macrophylla*,* Antiaris toxicaria*,* Millettia ferruginea*, and *Cordia africana* are dominant tree species of the study forest. In addition, *Albizia grandibracteata* and *Trichilia dregeana* contributes significant amount of basal area (BA) M^2^ ha^−1^, which has great implication to storing high amount of biomass and carbon (Daba and soromessa: Species composition, stand structure and regeneration status of tree species in Yayu coffee forest biosphere reserve, Illubabore zone, southwestern Ethiopia, unpublished).

### Species description

*Albizia grandibracteata* species belongs to the plant family Fabaceae; whereas, *T. dregeana* species belongs to the plant family Meliaceae. Both tree species have a wide range of ecological distributions [32]. *A. grandibracteata* species mostly grow in the upland rainforest and riverine forest areas, with preference in moist and wet sites. This tree species is known for its multiple provisional services [35]. It provides multiple socio-economic benefits (firewood, farm tools, medicine from the root, bee forage, ornamental, and soap from its bark); it is also known for nitrogen fixation which is one of its ecological services [32]. In Ethiopia, *A. grandibracteata* species grow in moist agroclimatic zones within an altitude range of 1200–1700 m asl. [33]. It is a medium-sized deciduous tree with a straight trunk to 20 m and flattened crown; it can also attain a maximum height of 30 m.

In Ethiopia, *T. dregeana* Sond. species occurs in the moist and wet montane rainforest of southwestern and eastern highland between 1100 and 2200 m asl. altitude ranges [33]. This tree species is well known for provisional services like firewood, timber (construction, furniture), and coffee shade under natural forest. The *T. dregeana *Sond. is a large evergreen tree grows up to 35 m height [36]. It has a straight trunk that can attain higher DBH size that divides into large branches and forms a rounded crown [33]. The selected tree species have a significant contribution in terms of stocking density and BA m^2^ ha^−1^; which indicates that these species have great potential in storing TAGB and carbon [37].

### Sampling method

The procedures of semi-destructive methodology in “Manual for building tree volume and biomass allometric equations” prepared by Food and Agriculture Organization (FAO) [38] were followed. A random sampling technique where all individuals have an equal probability of being involved in the study was applied. Therefore, the selection of individuals from different diameters at breast height (DBH) classes was used. The random sampling technique was conducted with a quick screening of vegetation variability across the landscape or particular locality; to delimit the vegetation type in mind [20]. The guideline by [10] suggests the relevance of considering some biophysical factors of the forest stand to have representative sample sets. Detail information about sampling trees, field-based measurements, and laboratory analysis can be found in [28, 38, 39].

### Tree biomass procedures

The biomass calculation has also followed the procedure in “Manual for building tree volume and biomass allometric equations” prepared by FAO [38]. During semi-destructive procedures for biomass, diameters of the trunk and large branches were measured at every 1 m length directly in the field. The fresh biomass of trimmed branches was also directly measured in the field. The volume and dry weight of the wood aliquots were measured in the laboratory which was later used for wood density calculation. Three small branches for every tree sampled (3 × 60 = 180) were trimmed for the determination of trimmed biomass. The fresh biomass of small untrimmed branches was calculated based on the relationship between BD and dry biomass of trimmed branches. The fresh biomass of large untrimmed branches and trunk was calculated from volume and wood density measurement. The tree sections were considered to be cylinder; whereas, density was considered to be the same for all compartments of the tree. The assumption is that along every 1 m length of a tree there is no tapering (variation in diameter is insignificant), and every section is considered to have cylinder shape [38].

### Measuring trimmed and untrimmed fresh biomass

The diameter at the bases of each trimmed branch was measured and the leaves of each branch were fully harvested from the wood. Only basal diameter was measured for small untrimmed branches. A section about one-meter length was preferred for diameter measurement of the trunk and main branches [40]. Detail information regarding measurement of trimmed branches in the field, measurement of wood and leaf aliquots in the laboratory, measurement of small and large untrimmed branches, and trunk can be found in [28, 38].

### Biomass calculations

All procedures of laboratory analysis related to wood and leaves aliquots and calculation of trimmed and untrimmed biomass can be found in the FAO manual [38] was used.

### Comparison between species-specific and pan-tropical allometric equations

The species-specific equation and pan-tropical allometric equations were compared in this study for accuracy assessment. The pan-tropical allometric equations potentially applicable in tropical moist forests were used for the comparison. In Ethiopia, these equations have been most frequently used for biomass estimation in the montane moist forest ecosystem. The datasets for biomass comparisons were generated form: (1) Measured biomass- generated based on semi-destructive procedures; (2) Specific Equation-equation which was developed for *A. grandibracteata* and *T. dregeana*; (3) Equations developed by [5, 12, 13, 41] for tropical forests.

### Data analysis and model selection

Points regarding the summarized data and statistical package used during the data analysis was can be found in [28]. Eight allometric equations were developed by evaluating the relationships among the considered variables i.e. TAGB against single predictor variables (D, H, ρ); TAGB against single compound variables (D^2^H, DH, ρDH); TAGB against multiple variables (D + H + ρ; D^2^H + ρ; D + H). These models were fitted based on log-transformed data and all have achieved model goodness of fit statistics. The relationship between TAGB and wood density was found statistically not significant (p > 0.05). Biomass regression models selections and evaluation were tested based on performance statistics including coefficient of determination (adj.R^2^), residual standard error (RSE), Akaike information criterion (AIC), and p-value. AIC is an estimator of the relative quality of statistical models for a given set of data. AIC estimates the quality of each model relative to each other [42]. All formulated models used natural logarithm transformation; to minimize the systematic bias during the back transformation a correction factor (CF) was calculated for each equation [43].

## Results

### Allometric equations and their performance

The allometric equations relating the dependent variable TAGB against the predictor variables (D, H, and ρ) were formulated for *A. grandibracteata* and *T. dregeana* tree species. The descriptive summary of these main variables for TAGB regression models formulation was presented in (Table 1).

**Table 1:** Descriptive summary of dendrometric variables for *A. grandibracteata* and *T. dregeana*

Tree species	Variables	Minimum	Maximum	Mean	Standard deviation
*A. grandibracteata*	TAGB	6.26	2268	587.1	629.17
	DBH	5.2	70.8	31.5	18.92
	H	4	38	22.97	11.14
	ρ	0.3559	0.5824	0.4709	0.0703
*T. dregeana*	TAGB	2.87	5502	939.6	1587.66
	D	5.2	105	36.37	28.94
	H	3.5	38	25.15	11.76
	ρ	0.2406	0.5799	0.4179	0.0745

*TAGB* aboveground biomass (in kg), *D* diameter at breast height (in cm), *H* total height (in m), *ρ* wood density (in g cm^−3^)

### The relationship between the predictor variables of *A. grandibracteata* and* T. dregeana*

**Fig. 2:**
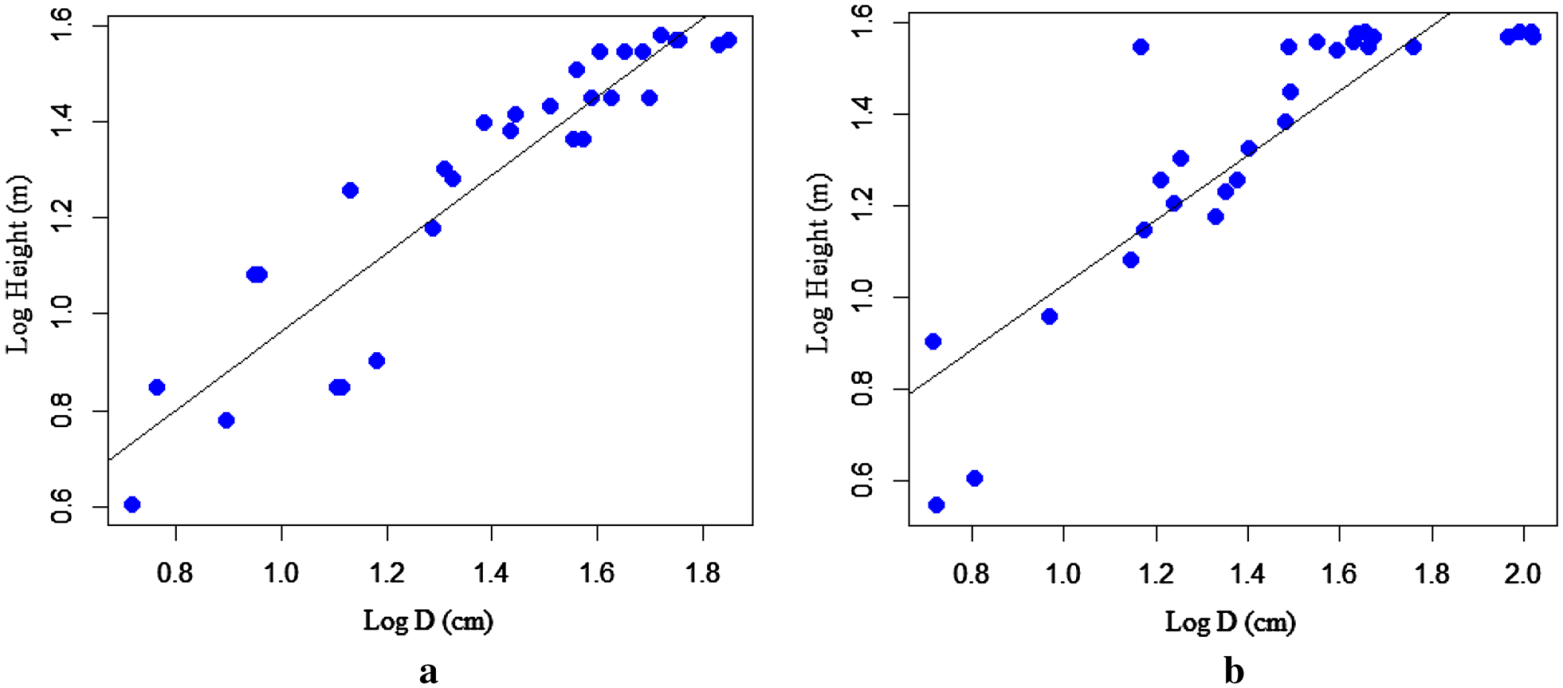
Scatter plot of Diameter-Height relationships for: **a** *A. grandibracteata*, **b** *T. dregeana* tree species

The correlation coefficient between the predictor variables was calculated using Pearson's correlation coefficient at a 95% confidence interval. Pearson's correlation coefficient is the statistical measure of the strength of a linear relationship between the paired variable (D & H, D & ρ). The correlation coefficient between D and H is 0.92 indicating a strong relationship between the variables for A. grandibracteata and also was found statistically significant at (p < 0.05). The correlation coefficient between D & H is 74.3, and statistically significant at (p < 0.05) for *T. dregeana*. However, the correlation coefficient between predictor variables (D & ρ) was found statistically not significant at (p > 0.05) for both tree species. Scatter plot depicted in (Fig. 2a, b) verifies the relationship between D & H of *A. grandibracteata* and *T. dregeana* specie*s* respectively; indicating an increment of tree height for a unit increment of its DBH. The DBH versus tree height plot was constructed for the area of interest; also, it has a purpose to compare and determine how appropriate the biomass regression model for a given site.

### Selected allometric equations

The allometric equations were formulated by relating TAGB against independent variables individually and in combination. The selected allometric equations were tested for goodness of fit based on different performance statistics. The coefficients for all selected allometric equations were found statistically significant (p ≤ 0.001); indicating the strong relationships between the TAGB and its predictor variables (Table 2).

**Table 2:** Best fitted regression models for predicting aboveground biomass of *A. grandibracteata* and *T. dregeana*

Equation No.	Allometric equations	Coefficients		Model performance statistics				
		Symbol	Value	Adj.R^2^	RSE	AIC	CF	p-value
AgEq1	TAGB = exp[α + β_1_ln(D) + β_2_ln(H) + β_3_ln(ρ)]	α	− 0.793	0.9936	0.1347	− 79.49	1.0091	≤ 0.001
		β_1_	2.117					
		β_2_	0.062					
		β_3_	0.991					
AgEq2	TAGB = exp[α + β_1_ ln(D^2^H) + β_2_ln(ρ)]	α	− 0.810	0.983	0.2198	− 50.96	1.0245	≤ 0.001
		β_1_	0.749					
		β_2_	1.030					
AgEq3	TAGB = exp[α + β_1_ln(D)]	α	− 1.744	0.9797	0.2408	− 46.41	1.0294	≤ 0.001
		β_1_	2.241					
AgEq4	TAGB = exp[α + β_1_ln(D) + β_2_ln(H)]	α	− 1.755	0.979	0.2449	− 44.49	1.0304	≤ 0.001
		β_1_	2.199					
		β_2_	0.049					
AgEq5	TAGB = exp[α + β_1_ln(D^2^H)]	α	− 1.834	0.9681	0.3016	− 32.89	1.0465	≤ 0.001
		β_1_	0.775					
AgEq6	TAGB = exp[α + β_1_ln(H)]	α	− 1.363	0.8545	0.6437	12.59	1.2302	≤ 0.001
		β_1_	2.286					
AgEq7	TAGB = exp[α + β_1_ln(ρDH)]	α	− 0.699	0.9682	0.3007	16.97	1.0462	≤ 0.001
		β_1_	1.129					
Ag Eq8	TAGB = exp[α + β_1_ln(DH)]	α	− 1.803	0.9514	0.3722	29.77	1.0717	≤ 0.001
		β_1_	1.172					
TdEq1	TAGB = exp[α + β_1_ ln(D) + β_2_ ln(H) + β_3_ ln(ρ)]	α	− 2.526	0.975	0.3204	22.55	1.0560	≤ 0.001
		β_1_	2.029					
		β_2_	0.593					
		β_3_	0.648					
TdEq 2	TAGB = exp[α + β_1_ln(D^2^H) + β_2_ln(ρ)]	α	− 2.756	0.973	0.3302	23.49	1.0560	≤ 0.001
		β_1_	0.897					
		β_2_	0.562					
TdEq 3	TAGB = exp[α + β_1_ln(D^2^H)]	α	− 3.168	0.972	0.3408	24.48	1.0598	≤ 0.001
		β_1_	0.888					
TdEq 4	TAGB = exp[α + β_1_ln(D) + β_2_ln(H)]	α	− 3.032	0.972	0.3371	24.74	1.0585	≤ 0.001
		β_1_	1.964					
		β_2_	0.641					
TdEq 5	TAGB = exp[α + β_1_ln(D)]	α	− 2.563	0.962	0.3911	32.74	1.0795	≤ 0.001
		β_1_	2.427					
TdEq 6	TAGB = exp[α + β_1_ln(DH)]	α	− 3.356	0.958	0.4121	35.87	1.0886	≤ 0.001
		β_1_	1.377					
TdEq 7	TAGB = exp[α + β_1_ln(ρDH)]	α	− 2.220	0.951	0.4467	40.71	1.1049	≤ 0.001
		β1	1.393					
TdEq 8	TAGB = exp[α + β_1_ln(H)]	α	− 3.088	0.819	0.8565	79.77	1.4431	≤ 0.001
		β_1_	2.771					

Where TAGB: aboveground tree biomass (kg); D: diameter at breast height of tree (cm); H: total tree height (m); ρ: Wood Density (g cm^−3^); α: intercept; β_1_, β_2_, β_3_: are slopes; adj.R^2^: adjusted R square; RSE: Residual Standard Error; AIC: Akaike Information Criterion; CF: correction factor; AgEq: *A. grandibracteata* Equation*;* TdEq:* T. dregeana* equation

### Scatter plots of TAGB against dendrometric variables for *A. grandibracteata*

**Fig. 3:**
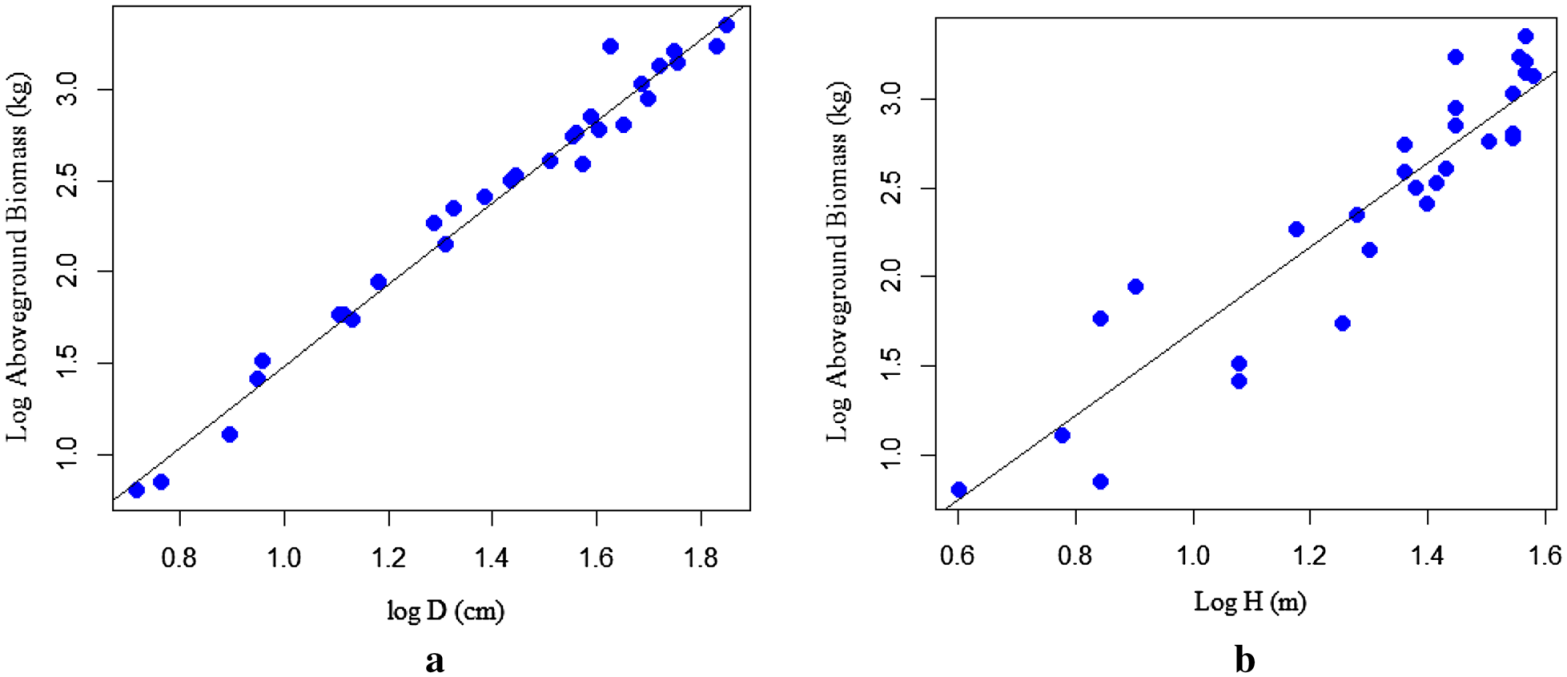
Linear regression for log-transformed data: **a** aboveground biomass against D; **b** aboveground biomass against height

**Fig. 4:**
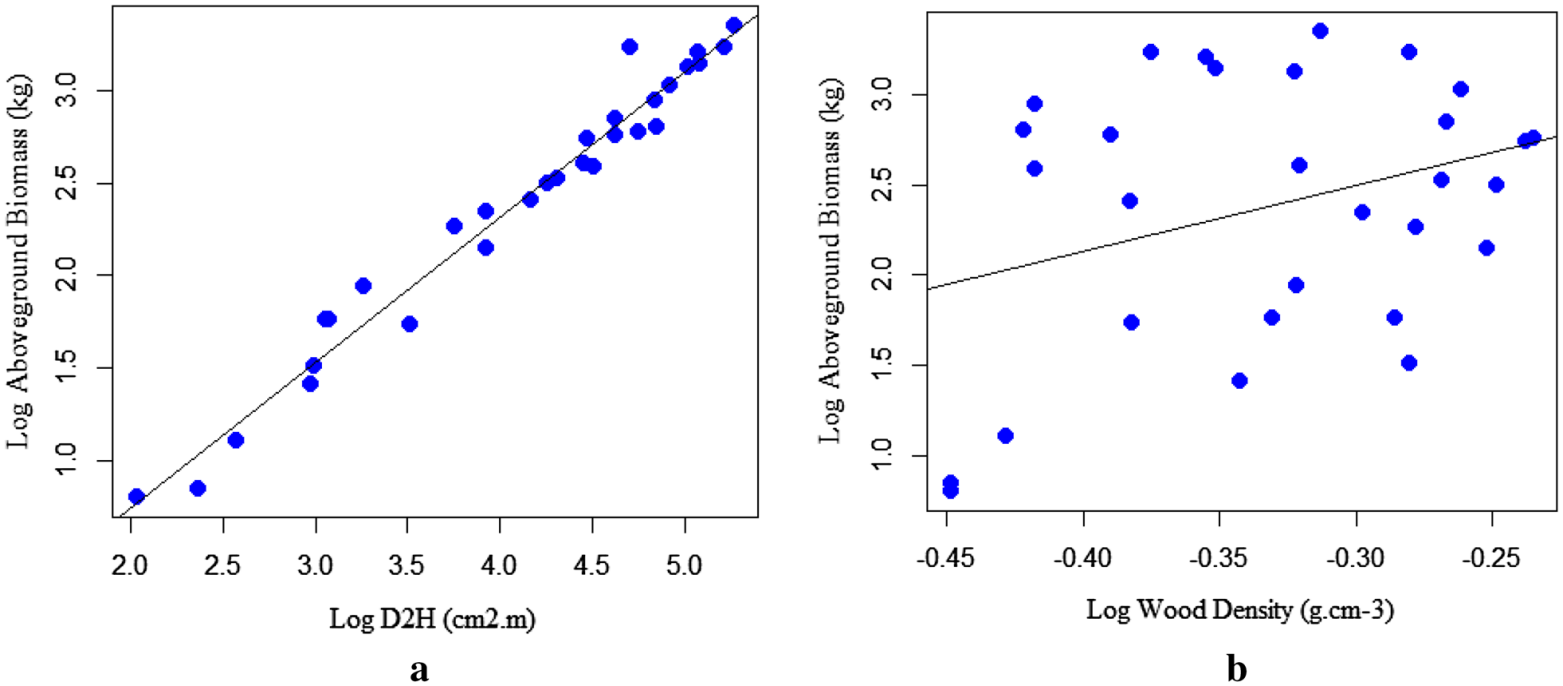
Linear regression for log-transformed data: **a** aboveground biomass against D^2^H, **b** aboveground biomass against wood density

As depicted in (Fig. 2b, 3a, and 4a) that the dependent variable (TAGB) increases with a unit increase of independent variables (D, H, and D^2^H). This indicates the existence of a strong relationship between TAGB and the predictive variables. However, a significant relationship was not observed between TAGB and ρ (Fig. 4b). The relationship between wood density and tree dendrometric variables is quite complex for a tree species related to microsite variation and tree maturity as well as due to different site factors.

### Scatter plots of TAGB against dendrometric variables for *T. dregeana*

**Fig. 5:**
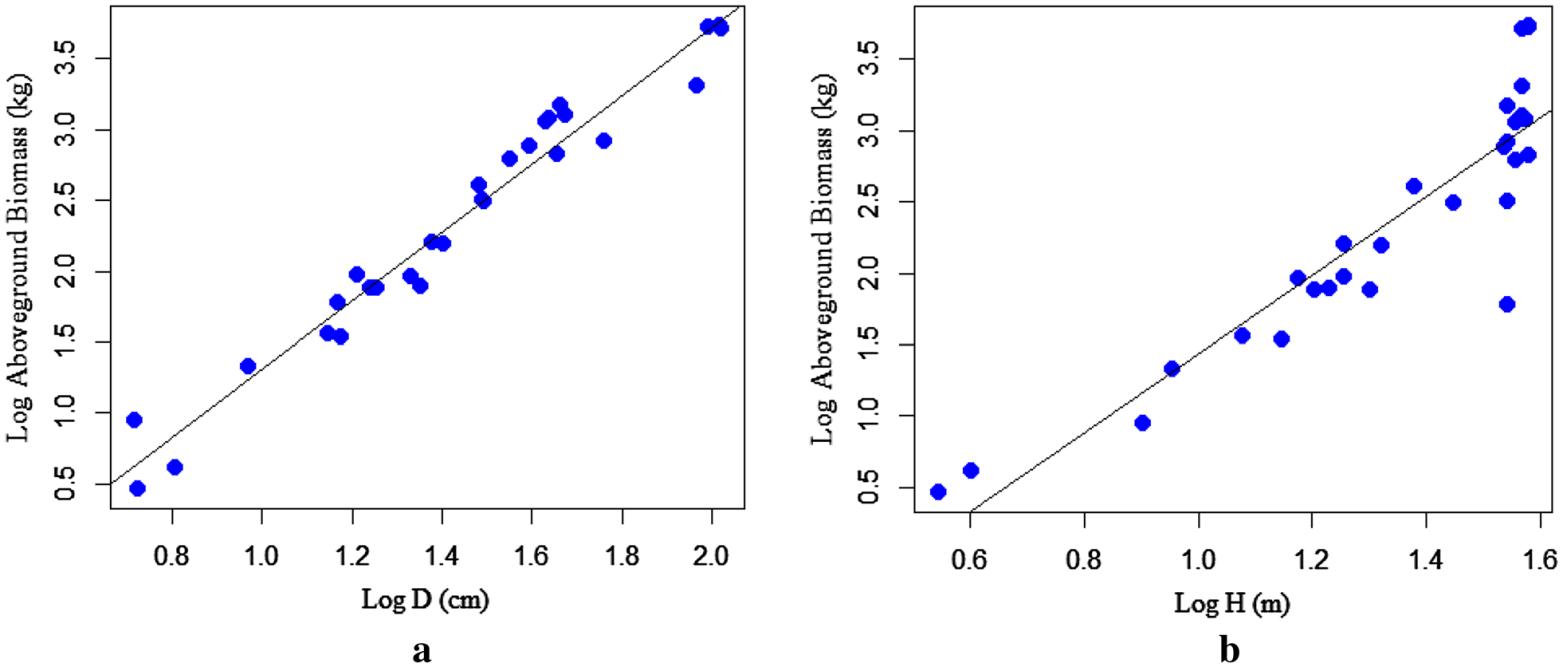
Scatter plots for: **a** aboveground biomass against diameter, **b** aboveground biomass against height for *T. dregeana*

**Fig. 6:**
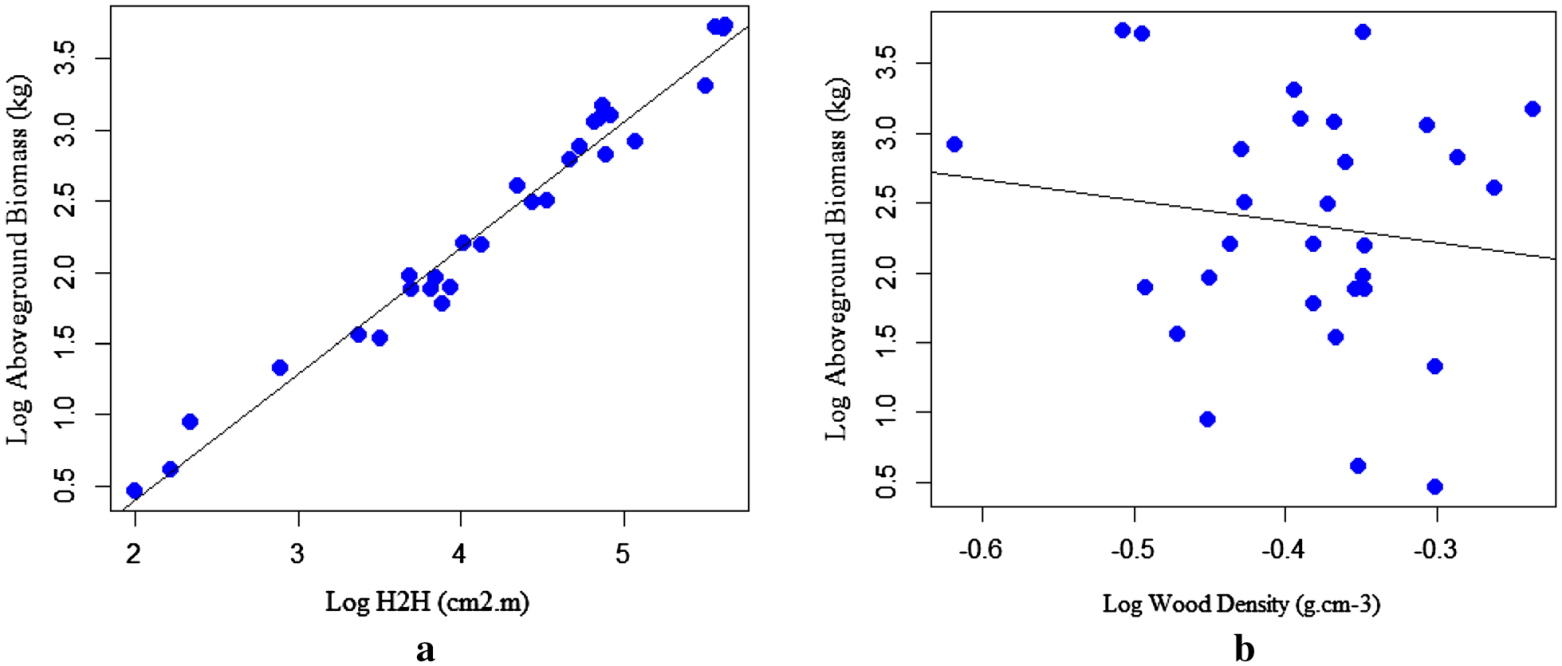
Scatter plots for: **a** TAGB against D^2^H, **b** TAGB against wood density for *T. dregeana*

The scatter plots displayed in (Fig. 5a, b and 6a) shows the increase of TAGB against a unit variation of the predictor variables (D, H, & D^2^H). The scatter plots of TAGB against D exhibits the linear relationship. The relationship between TAGB and H also shows an increment of TAGB for an increment of tree height. However, the relationship between TAGB and ρ has shown a weak association, due to the complex relationship of wood density with site factors and stand structure.

The scatter plot of TAGB against D^2^H shows linearity; however, a significant relationship was not observed between TAGB and ρ as displayed in (Fig. 5).

### Selected biomass regression models for *A. grandibracteata*

The species-specific biomass regression models were developed for the selected tree species based on log-transformed data. All selected allometric equations have achieved the model goodness of fit. The best performing regression models of *A. grandibracteata* and *T. dregeana* were listed in decreasing order of importance based on AIC value (Table 2).

The formulated allometric equations were listed in decreasing order of importance from (AgEq1–AgEq8 and TdEq1–TdEq8) respectively for *A. grandibracteata* and *T. dregeana* to estimate TAGB (Table 2). The equations were ordered based on their AIC values, where the lower the AIC vale comparatively is the best equation and vice versa. Therefore, comparisons among the selected allometric equations of *A. grandibracteata* shows that AgEq1 was found best based on its AIC (− 79.49) value. This model shows strong relationships between the TAGB and the main predictive variables. The value of adj. R^2^ describes that 99.36% variation in TAGB was explained by the predictor variables in this model. The 2nd best-performing equation was (AgEq2) which was formulated by relating TAGB against D^2^H and wood density. It is the biomass regression model with a lower AIC value (− 50.96) based on statistical criteria for model selection. A strong relationship was observed between TAGB and the predictive variables. The value of adj. R^2^ also describes that the predictor variables explain 98.30% variation of the TAGB.

The 3rd best equation (AgEq3) was formulated by relating TAGB with D. The adj. R^2^ value for this biomass regression equation has shown (97.97%); indicating that D is a single tree dendrometric variable that best explains variation in TAGB of *A. grandibracteata* species. Other selected equations (AgEq4, 5, 6, 7, and 8) have also achieved models performance statistics and also listed in decreasing order of importance based on AIC value (− 44.49, − 32.89, 12.59, 16.97, 29.77) respectively. There is a lower value of adj.R^2^ and a higher value of RSE for these models compared to the above models (1, 2, and 3). Generally, a strong relationship was found between TAGB and the dendrometric variables for all models. However, H has explained (adj. R^2^ = 81.9%) variation in TAGB of *A. grandibracteata* tree species. TAGB against predictive variable (H) has achieved a strong relationship which is statistically significant at (p ≤ 0.001). In contrast, a model relating TAGB with ρ was found statistically insignificant and then rejected.

*Trichilia dregeana* biomass regression model (TdEq1) was found as the best model with the least AIC value (22.55). The predictor variables in this model have explained 97.5% of the variation in TAGB. Also, the lower value of RSE is the parameter that has proved the fitness of this model. The allometric equation (TdEq2) has also shown the model goodness of fit well among the selected biomass regression models. This model predicts the relationship of TAGB against the main variables (D^2^H + ρ) and each predictive variable was independently fitted. The predictive variables explain adj.R^2^ (97.3%) variation of TAGB for this tree species. The relationship between TAGB and the predictor variable was also found highly significant at (p ≤ 0.001).

The other equations (TdEq3, 4, 5, 6, 7, & 8) were listed in decreasing order of importance considering their AIV values (24.48, 24.74, 32.74, 35.87, 40.71, 79.71) respectively. Generally, these models have achieved the goodness of fit statistics considering the adj.R^2^, RSE, & AIV values. The single predictive variable D in (TdEq5) explains adj.R^2^ (96.2%) variation in TAGB. Tree diameter is among the dendrometric variable that can be accurately and easily measured in the field. However, tree height (H) as a single predictor variable in TdEq8 was found to explain adj. R^2^ (81.9%) of TAGB variation which is lowest compared to all other formulated equation. The highest value of RSE (0.8565) was also recorded for this equation when compared to the other selected models.

### Comparison of species-specific with pan-tropical equations

The pan-tropical equations that have been used for estimation of forest biomass and carbon stocks in Ethiopia were compared with species-specific equations of *A. grandibracteata* and *T. dregeana*. Summary of the statistical parameters (paired t-test, mean difference of TAGB, percent bias, and root mean square error) for equations comparison was presented in (Table 3). The statistical parameters calculated were based on observed and predicted TAGB. The species-specific equation found better in accurately predicting TAGB of *A. grandibracteata* and *T. dregeana.* When compared to pan-tropical equation. The higher value of PBIAS shows the poor performance of pan-tropical equations in predicting TAGB of a specific species. This proofs the application of species specific allometric equation is fundamental to accurately estimate TAGB of tree species.

**Table 3:** Comparison of species-specific to pan-tropical equations in predicting biomass of *A. grandibracteata* and *T. dregeana*

Input variable	Source	Type	Equations	Mean biomass difference (kg)	PBIAS	RMSE	Pared t-test	
							T-value	p-value
D	Chave et al. [12]	PT	AGB = ρ × 0.223 × (D)^2.148^ × (D^2^)^0.207^ × (D^3^)^0.028^	1117.20	190.29	1859.62	4.047	0.000
	AgEq3	SS	TAGB = 0.175 × D^2.241^	6.45	1.09	213.94	0.163	0.872
D, H, ρ	Chave et al. [5]	PT	TAGB. = 0.0673 × (ρD^2^H)^0.976^	457.88	77.99	776.72	3.931	0.000
	AgEq2	SS	TAGB = 0.445 × (D^2^H)^0.749^ × ρ^1.030^	63.21	− 10.77	216.44	− 1.644	0.111
	AgEq5	SS	TAGB = 0.159 × (D^2^H)^0.787^	41.99	− 7.15	212.58	− 1.085	0.287
	AgEq7	SS	TAGB = 0.497 × (ρDH)^1.129^	95.61	− 16.29	269.68	− 2.042	0.050
	Brown et al. [41]	TFM	TAGB = 0.0899 × (D^2^Hρ)^0.9522^	499.95	85.16	822.14	4.125	0.000
	AgEq1	SS	TAGB = 0.452 × D^2.117^ × H^0.062^ ×ss ρ^0.991^	33.85	− 5.77	179.34	− 0.035	0.309
D	Brown [13]	TFM	TAGB = 0.118 × D^2.53^	1235	210.36	2073.59	3.993	0.000
	AgEq3	SS	TAGB = 0.175 × D^2.241^	6.45	1.09	213.94	0.163	0.872
D, H, ρ	Chave et al. [12]	TFM	AGB = 0.0509 × ρD^2^H	430.28	73.29	752.12	3.756	0.001
	AgEq1	SS	TAGB = 0.452 × D^2.117^ × H^0.062^ × ρ^0.991^	33.85	− 5.77	179.34	− 0.035	0.309
	AgEq4	SS	TAGB = 0.173 × D^2.199^ × H^0.049^	3.6292	0.62	211.03	0.093	0.927
	AgEq2	SS	TAGB = 0.445 × (D^2^H)^0.749^ × ρ^1.030^	63.21	− 10.77	216.44	− 1.644	0.111
	AgEq7	SS	TAGB = 0.497 × (ρDH)^1.129^	95.61	− 16.29	269.68	− 2.042	0.050
D	Chave et al. [12]	PT	TAGB = ρ × 0.223 × (D)^2.148^ × (D2)^0.207^ × (D3)^0.028^	1990.50	211.85	4322.19	− 2.794	0.009
	TdEq 5	SS	TAGB = 0.077 × D^2.427^	89.89	9.57	552.83	− 0.889	0.381
D,H, ρ	Chave et al. [5]	PT	TAGB. = 0.0673 × (ρD^2^H)^0.976^	555.82	59.15	1099.35	− 3.156	0.004
	TdEq2	SS	TAGB = 0.064 × (D^2^H)^0.897^ × ρ^0.562^	130.80	− 13.92	575.44	1.257	0.219
	TdEq3	SS	TAGB = 0.042 × (D^2^H)^0.888^	124.93	− 13.29	534.76	1.294	0.206
	Brown et al. [41]	TFM	TAGB = 0.0899 × (D^2^Hρ)^0.9522^	592.60	63.07	1128.80	− 3.322	0.002
	TdEq1	SS	TAGB = 0.0799 × D^2.029^ × H^0.593^ × ρ^0.648^	85.33	− 9.08	493.59	0.945	0.352
	TdEq2	SS	TAGB = 0.064 × (D^2^H)^0.897^ × ρ^0.562^	130.80	− 13.92	575.44	1.257	0.219
	TdEq7	SS	TAGB = 0.109 × (ρDH)^1.393^	236.08	− 25.13	907.31	1.451	0.157
D	Brown [13]	TFM	TAGB = 0.118 × D^2.53^	1532.30	163.08	3409.18	− 2.709	0.011
	TdEq5	SS	TAGB = 0.077 × D^2.427^	89.89	9.57	552.83	− 0.889	0.381
D, H, ρ	Chave et al. [12]	TFM	TAGB = 0.0509ρD^2^H	539.11	57.38	1101.95	− 3.021	0.005
	TdEq1	SS	TAGB = 0.0799 × D^2.029^ × H^0.593^ × ρ^0.648^	85.325	− 9.08	493.59	0.945	0.352
	TdEq2	SS	TAGB = 0.064 × (D^2^H)^0.897^ × ρ^0.562^	130.80	− 13.92	575.44	1.257	0.219
	TdEq3	SS	TAGB = 0.042 × (D^2^H)^0.888^	124.93	− 13.29	534.76	1.294	0.206
	TdEq7	SS	TAGB = 0.109 × (ρDH)^1.393^	236.08	− 25.13	907.31	1.451	0.157

PT: Pan-tropical, TFM: Tropical Forests Moist, SS: Species-specific, PBIAS: percent bias, RMSE: root mean square error, D: diameter at breast height, H: total height, ρ: wood density, AgEq: *A. grandibracteata* Equation; TdEq:* T. dregeana* Equation

**Fig. 7:**
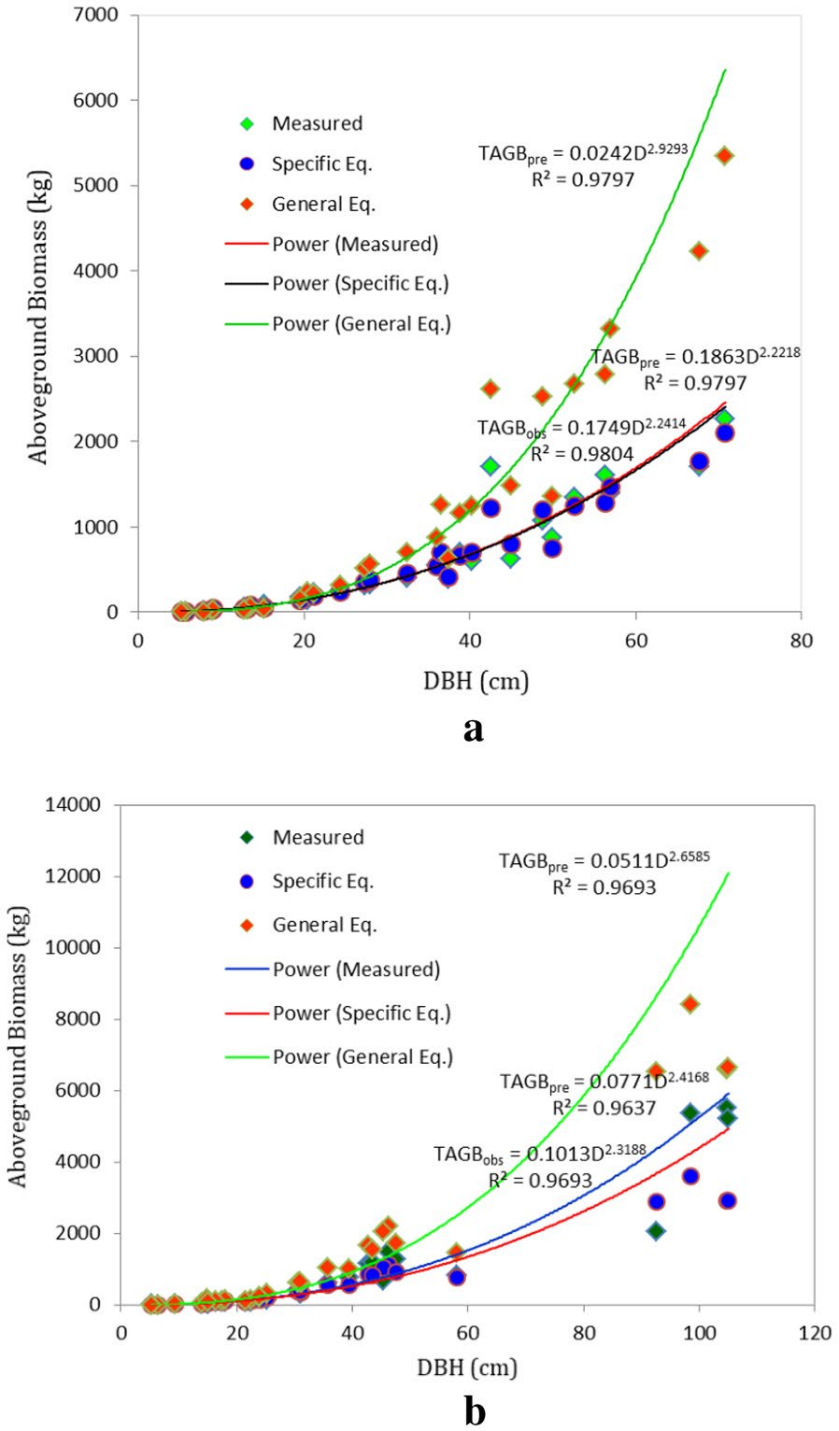
Species specific and pan-tropical allometric equations comparison for: **a** *A. grandibracteata,* **b** *T. dregeana* TAGB. Measured biomass: was obtained based on the semi-destructive methodology for allometric equation; Specific Equation: equation which was developed for *A. grandibracteata* and *T. dregeana*; Generalized Equation: was taken from [12] for pantropical tropical moist forest stands

The allometric equation TAGB = 0.0509(ρD^2^H) by [12] is potentially applicable equation in tropical moist forests. In addition, this equation has been most frequently used for biomass estimation in Afromontane rainforests of Ethiopia. Species-specific equation with input variable (ρD^2^H) was formulated (i.e. TAGB = 0.3274 × (ρD^2^H)^0.759^ for *A. grandibracteata* and TAGB = 0.0832 × (ρD^2^H)^0.899^ for *T. dregeana*) based on measured dataset of total aboveground biomass. Therefore, Chave’s equation was compared with the species-specific equation as depicted in (Fig. 7a, b).The power models were plotted by regressing tree DBH against TAGB (Measured and predicted biomass). The predicted TAGB was obtained using species-specific equations (Species Eq.) and generalized equation (Generalized Eq.).

## Discussion

The biomass regression model formulated by relating TAGB against multiple variables (D, H, and ρ) was found statistically the best performing equation for the selected regression models of *A. grandibracteata* and *T. dregeana* tree species. This model has considered important predictive variables that improve the accuracy of estimating TAGB. Several studies also suggest the importance of considering the dendrometric variables (D, H, and ρ) in formulating biomass regression models [25, 44]. Studies explain that TAGB estimation is inaccurate when tree height is not available as the predictor variable. Allometric equations are more likely to vary across vegetation types since diameter and height are influenced by the environmental condition.

The compound variable of diameter and height (D^2^H) as a single predictor or in combination with ρ is robust in prediction TAGB of the tree in this study. Allometric equations with such predictor variables are mostly proposed for its wide range of applications. The study of [45] also reports that the combination of predictor variables (D & H) is used to capture volume variation. The important predictor variables (D & H) can be directly modulated with climatic and physiographic factors, hence affect the biomass. For instance, the biomass regression model that relates TAGB against the compound variable (ρD^2^H) was found the best fit model in many studies [5, 12].

Wood density is the best dendrometric variable that converts tree volume into biomass. However, A significant relationship was not established when data of TAGB was regressed against the wood density of both *A. grandibracteata* and *T. dregeana.* Generally, wood density varies among individuals of the same tree species due to the variability of environmental conditions. Similarly, the study of [46] explains the variation of specific wood gravity of a tree species with spatial variation across a forest landscape. Also, the comprehensive study of [47] reveals the variation of tree wood density is most likely correlated with increasing elevation, the coarseness of soil texture, and drought stress. The importance of wood density as a single variable in yielding the best fitted TAGB model is a great point of debate in several recent studies. The comprehensive study of [48] explains that xylem density which is the physical property of wood varies between individuals, species, and environments. Since, it reflects the physiological strategies of trees that lead to growth and survival, wood density as a single predictor variable will not be correlated with TAGB. Several other studies confirm the regional variation of stand-level wood specific gravity that can significantly affect the variation of TAGB. Overall, the great importance of wood density is reported in carbon accounting of tropical forests. However, its variation among different species is correlated with morphological, mechanical, physiological and ecological properties [21, 49].

The biomass regression model relating TAGB against D has achieved the model goodness of fit and found statistically significant in this study. This has confirmed that there is a strong relationship between TAGB and D. Basically, D measurement is accurate and practical when compared to other dendrometric variables. Several studies have also reported the significance of D in predicting TAGB [9, 12, 50]. In this study, many of the biomass regression models formulated for TAGB against predictors (DH) or (D + H) were also found the best performing models. These equations are robust in predicting TAGB of the tree species considered in the study. Similar studies also clarify that the addition of diameter and height as a predictive variable in biomass measurement shows improvement in the TAGB variation.

Also, such models have anticipated to increases the accuracy at a multiregional scale [20, 51] related to its application. On the other hand, the relationship between D and H is modulated by multiple environmental factors of forests [52, 53]. The report of [5] also suggests the consideration of the diameter-height relationship for locally developed allometric equations. The ultimate reason is that the variation of the relationship between these predictive variables depends directly on the bioclimatic variables.

The existence of a few allometric equations for sub-Saharan Africa is reported by several studies [10, 54, 55]. Many of adopted generalized equations generate great uncertainty of biomass. The study of [14] reports that higher bias was observed related to the Chave’s model II largely overestimating by approximately 300% to 400% for two tropical forest sites. This confirms the significance of formulating species-and site-specific allometric equations for tropical forests. Such an approach avoids a systematic error generated related to the generalized equation which possibly propagates to the national and global carbon budget. Generally, in response to global climate change mitigation, the monitoring and assessment of carbon dioxide from forests is essential. Ethiopia is known for its diverse vegetation ecosystems and associated high diversity of woody plants. However, the assessment of biomass and carbon stock of forests has been practiced by adopting the generic pan-tropical allometric equations that cause great uncertainty. Therefore, the development and application of species-specific allometric equation is inevitable for accurate estimation of biomass. Formulating allometric equations for all woody plants in Ethiopia is quite desirable for accurately quantifying the biomass and carbon stock of forests to achieve accurate national and international reporting of carbon dioxide emission inventories.

## Conclusions

Formulating an allometric equation is an important approach for the estimation of tree biomass. It has also an indirect role in contributing to the assessment and monitoring of the global carbon cycle. Adopting the generic pantropical allometric equation has the limitation of uncertainty in quantifying biomass of specific forest stand. This is a particularly serious problem for the assessment of carbon stock in tropical forests of Africa, related to ecological variability and diverse tree species. Species-specific allometric equations were formulated for *A. grandibracteata* and *T. dregeana* species following the procedure of semi-destructive methodology. The formulated equations are proposed as a species-specific equation particularly in the Afromontane rainforest as well as in the montane moist forest ecosystem of southwestern Ethiopia.

## Data Availability

The datasets used and/or analyzed during the current study are available from the corresponding author on reasonable request.
